# Specific Functions of BIG1 and BIG2 in Endomembrane Organization

**DOI:** 10.1371/journal.pone.0009898

**Published:** 2010-03-25

**Authors:** Frédéric Boal, David J. Stephens

**Affiliations:** Cell Biology Laboratories, Department of Biochemistry, University of Bristol School of Medical Sciences, Bristol, United Kingdom; Institut Européen de Chimie et Biologie, France

## Abstract

**Background:**

Transport of molecules from one subcellular compartment to another involves the recruitment of cytosolic coat protein complexes to a donor membrane to concentrate cargo, deform the membrane and ultimately to form an independent carrier. Small-GTP-binding proteins of the Arf family are central to many membrane trafficking events. Arfs are activated by guanine nucleotide exchange factors (GEFs) which results in their recruitment to membranes and subsequent engagement with Arf-effectors, many of which are coat proteins. Among the human BFA-sensitive large Arf-GEFs, the function of the two closely related BIG1 and BIG2 is still not clear, and recent studies have raised the question of functional redundancy between the two proteins.

**Methodology/Principal Findings:**

Here we have used small-interfering RNA on human cells and a combination of fixed and live-cell imaging to investigate the differential functions of BIG1 and BIG2 in endomembrane organization and function. Importantly, in this direct comparative study, we show discrete functions for BIG1 and BIG2. Our results show that depletion of BIG2 but not of BIG1 induces a tubulation of the recycling endosomal compartment, consistent with a specific role for BIG2 here. In contrast, suppression of BIG1 induces the formation of Golgi mini-stacks still polarized and functional in terms of cargo export.

**Conclusions:**

A key finding from our work is that suppression of BIG1 expression results in a fragmentation of the Golgi apparatus. Our data indicate that the human BFA-sensitive large Arf-GEFs have non-redundant functions in cell organization and membrane trafficking. BIG1 is required to maintain the normal morphology of the Golgi; BIG2 is important for endosomal compartment integrity and cannot replace the function of BIG1 in Golgi organization.

## Introduction

The transport of proteins and lipids between different compartments of the secretory pathway involves the budding of a coated vesicle from a donor compartment. This process involves the selection and incorporation of a cargo protein into nascent vesicles, followed by scission from the donor compartment, release of the coat, and subsequent transport of the vesicle to the acceptor compartment. Membrane fusion completes the transfer of cargo to the acceptor compartment [1]. Several coat complexes are recruited in different sub-compartments: the COPII machinery is recruited on the endoplasmic reticulum exit sites (ERES) and directs cargo export from the ER and transit to the ER-Golgi intermediate compartment (ERGIC). Subsequent transport steps require the COPI machinery, believed to regulate both anterograde and retrograde trafficking between the Golgi and the ERGIC [2]. Transport at the TGN boundary includes the formation of clathrin-coated vesicles where clathrin is recruited by different adaptor proteins including the multimeric AP-1, AP-3 and AP-4 [3] as well as the monomeric gamma ear Golgi-localized Arf-binding proteins (GGAs) [1].

The formation of coated vesicles depends on the tightly controlled activation of several small GTP-binding proteins. As Sar1 initiates the recruitment of COPII [4], the ADP-ribosylation factors (Arfs) recruit the other coats [5]. Mammalian Arfs are subdivided into three classes according to sequence homology: class I (Arf1, 3), class II (Arf4, 5), and the sole known member for class III Arf6 [5]. Arfs act as molecular switches, cycling between an inactive GDP-bound state and an active GTP-bound state. GTP-bound Arfs specify the recruitment of downstream effectors including these adaptors and therefore Arf activation is critical to the core mechanism and fidelity of membrane traffic. Arf activation through GDP-GTP exchange is mediated by guanine nucleotide exchange factors (GEFs) [6], [7].

Arf-GEFs are characterized by the catalytically active conserved Sec7 domain, and can be divided into two large families: the low-molecular-weight GEFs (<100 kDa) and the high-molecular weight GEFs (>100 kDa) [6], [7], [8]. The low-molecular-weight GEFs are not found in the yeast, suggesting a function specific to higher eukaryotes. This family contains in human ARNO, cytohesin-1, GRP1/ARNO3 and EFA6. Several studies suggest that these small GEFs are mainly involved in signal-transduction pathways originating to the cell surface or clathrin-dependent endocytosis, mainly by activation of the class III Arf6. The high-molecular-weight GEFs have orthologues in all eukaryotes investigated, suggesting evolutionary-conserved functions in membrane trafficking. The three human large Arf-GEFs, known as GBF1, BIG1, and BIG2, are all sensitive to the fungal metabolite brefeldin A (BFA). BFA inhibits the secretory pathway by inducing the release of coat complexes from the membranes, a collapse of the Golgi apparatus to the ER, a tubulation of the TGN and merging of the TGN with endosomal compartments [9], [10], [11], [12].

GBF1 acts at the ER/Golgi interface and within the Golgi stacks. GBF1 directs the assembly of COPI onto membranes and plays a key role in transport to and through the Golgi (for examples see [13], [14], [15], [16]). Less is known about the functions of BIG1 and BIG2 which were initially characterized as part of the same macromolecular complex (>600 kDa) [17]. BIG1 colocalizes with clathrin and AP-1 [17] and to a lesser extent with TGN38, but not with COPI [18]. In serum-starved cells, BIG1 can translocate into the nucleus [19] suggesting a role in a stress-induced signaling pathway. Silencing of BIG1 expression induces a minor disruption of the Golgi complex, described as a looser Golgi, and a profound defect in the maturation of β1-integrin followed by a subsequent defect in the spreading of the cells [20].

BIG2 has been shown to colocalize with the adaptor complex AP-1 [17] on the TGN, and with GGA3 and the cation-independent mannose-6-phosphate receptor (CI-MPR) [21], [22]. It does not colocalize with early- or late-endosomal markers such as EEA1 and LAMP1 [22], but seems to be involved in the maintenance of the endosomal compartment integrity and in the recycling of the transferrin [21], [23]. Mutations in BIG2 are found in genetic disorders leading to microcephaly and periventricular heterotopia [24], highlighting important and specific functions of BIG2 in humans. The lack of defects in tissues outside of the central nervous system is indicative of redundancy with other large Arf-GEFs. BIG1 and BIG2 have both been characterized as protein kinase A-anchoring proteins (AKAP) [25]. Furthermore, their phosphorylation by PKA decreases their GEF activity and induces their translocation to membranes, suggesting a possible role of BIGs in crosstalk between Arf and PKA pathways [25], [26].

While there have been several studies of large Arf-GEF function exploiting the power of RNAi, data from these studies are conflicting and often assume complete redundancy between BIG1 and BIG2 in particular. In this study, we have performed siRNA-mediated depletion of BIG1 and BIG2 in human cells to better understand their individual functions.

## Results

### Depletion of BIG1 or BIG2

**Figure 1:**
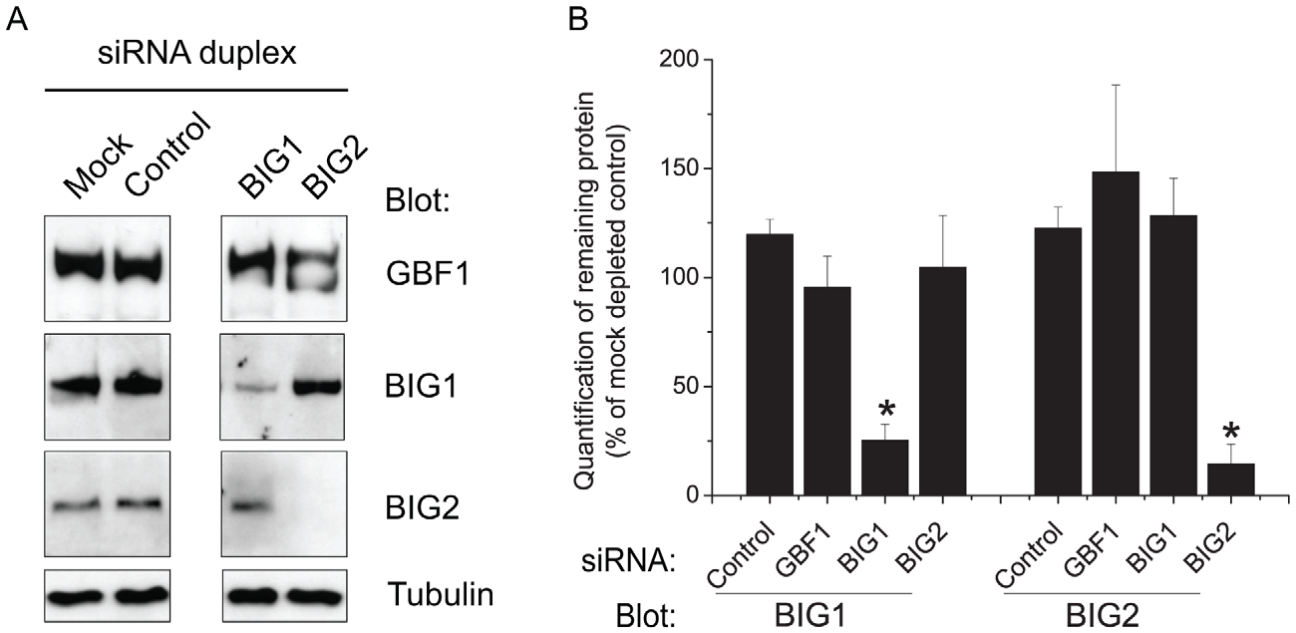
Depletion of human large-GEFs using siRNA. **A:** HeLa cells were transfected without siRNA (Mock), with non-targeting siRNA duplexes (Control), or with siRNA targeting indicated GEF (GBF1, BIG1, BIG2). Cell lysates were then immunoblotted for antibodies indicated on the left (Blot). Arrowhead points to the specific band for BIG1. **B:** Western-blots were quantified by densitometry and protein contents are expressed as percentage of mock-transfected cells. Results are presented as mean +/− s.e.m., n = 3–4, asterisk means p<0.05 as compared to cells transfected with a non-targeted siRNA (Control). Molecular-mass markers are shown in kDa in all figures.

To determine the individual functions of the large Arf-GEFs, we depleted these proteins from cells using RNA interference. Immunoblotting with antibodies directed against each of these proteins showed that we were able to deplete them with a pool of two different duplexes after 72 hours of transfection (Fig. 1A). The expression level of GBF1 remains unaffected and duplexes targeting BIG1 do not reduce the levels of BIG2 and vice versa. The quantification by densitometry from 3 to 4 independent experiments showed that BIG2 was depleted to 86% and BIG1 to 75% across the overall cell population (Fig. 1B).

### BIG2 is needed for the endosomal compartment integrity

**Figure 2:**
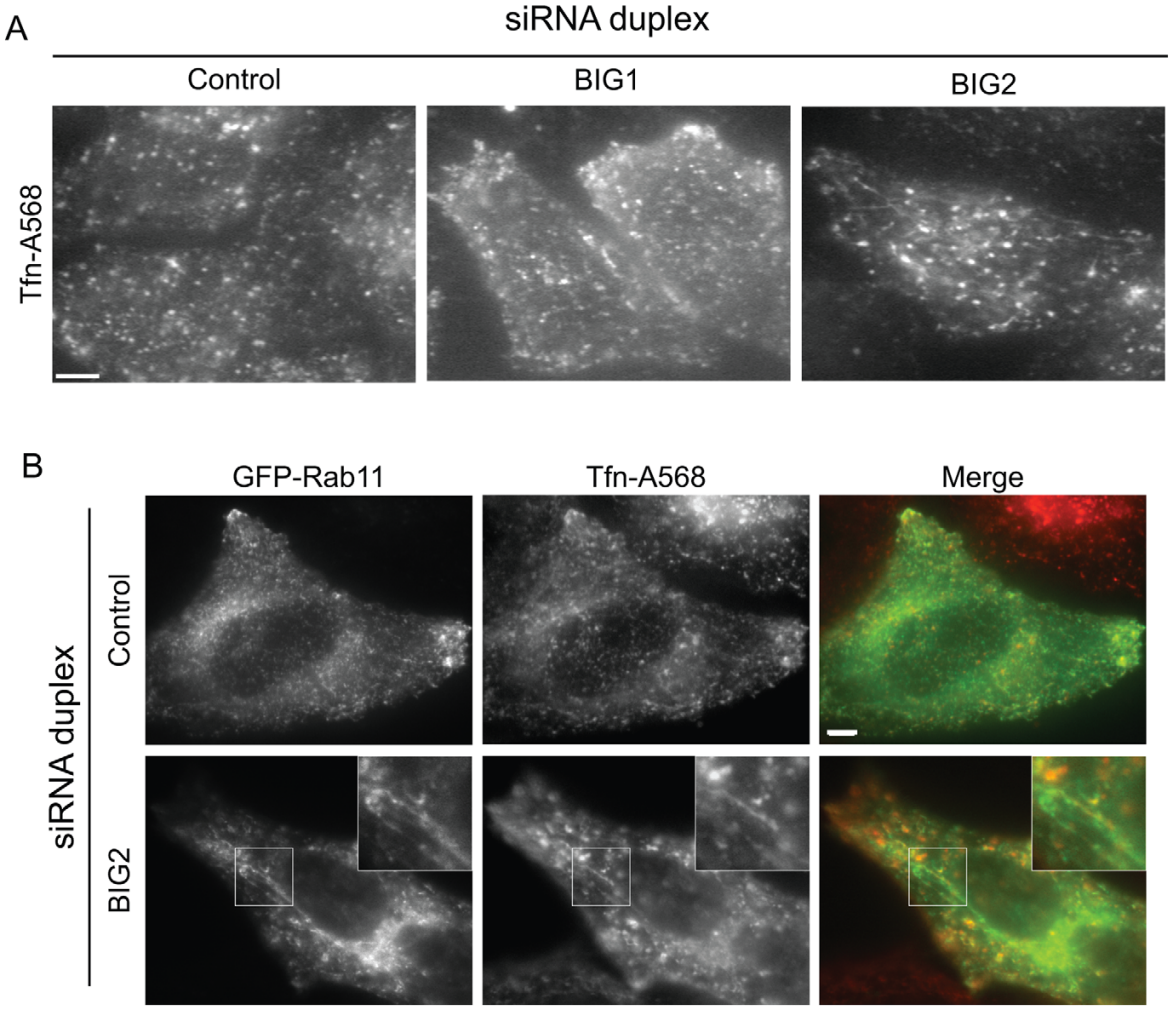
Depletion of BIG2 induces the tubulation of the endosomal compartment. **A:** HeLa cells depleted using siRNA duplexes as indicated were loaded with fluorescent transferrin (Tfn-A568) for 30 minutes prior to live-cell imaging. Movie S1 shows the stability of the BIG2-depletion induced tubules. **B:** Tfn-A568 loaded cells were co-labelled using GFP-Rab11. Single optical z-sections are shown resulting in the presence of some out-of-focus light, and panels below show enlarged regions. Bar = 10 µm in all figures otherwise stated.

In order to determine the effect of these depletions on the gross morphology of different subcellular compartment, we performed immunofluorescence on depleted cells with antibodies specific for several different organelles. First, the depletion of BIG1 or BIG2 had no effect on ERES (as revealed by COPII labelling, Fig. S1, COPII), or late endosomes/lysosomes (CD63 labelling, data not shown). BIG2 has been proposed to act at the TGN-endosome boundary [18], [21], [27]. Two different groups have suggested a role of BIG2 in the recycling of transferrin, and its depletion induces an accumulation of uploaded transferrin and its receptor in the juxtanuclear region [21], [23]. BIG2 depletion seemed not to have an effect on the gross morphology or distribution of early endosomes (EEA1 labelling, Fig. S1, EEA1). Transferrin bound to its receptor is believed to move sequentially to early-endosomes, then to recycling-endosomes (REs) and subsequently is recycled to the cell surface. The half-life of these events has been measured to be between 5 minutes (if transferrin is recycled independently of the REs) or 15–30 minutes (through REs) [28], [29]. In order to investigate the effect of BIG2-depletion on endosomal compartments, we imaged live-cells after a 30 minutes pulse of fluorescent transferrin. As shown in Fig. 2A, BIG2-depletion induced an extensive tubulation of the endosomal compartment. Quantification shows that in BIG2-depleted cells, 70% of cells show tubulated transferrin-positive endosomes. This is consistent with previously published data [21], [23]. Recycling endosomes are believed to be a tubulo-vesicular organelle [30], but such an extensive tubulation was never observed either in control cells or in BIG1-depleted cells. Tubules observed in BIG2-depleted cells appeared to be very stable over time (see Movie S1) and vesicles were seen to move apparently alongside these tubules. These vesicles are probably early-endosomes or transport intermediates, known to associate with microtubules [31]. This suggests that these tubules themselves elongate alongside the microtubules, as it has been described for BFA-induced endosomal tubules [32], [33]. We found that only a subpopulation of these tubules is preserved upon fixation, precluding any further analysis by standard immunofluorescence techniques. Nevertheless, as shown in Fig. S2A, the microtubule network seemed to be completely unaffected by BIG2-depletion.

Previous work using overexpression of dominant negative mutants of BIG2, which also tubulates the endosomal network, have shown that Rab11 and Rab4 associate with these tubulated compartments [21], [34]. Using live cell imaging, we find that BIG2 depletion induces transferrin-positive tubules that label with GFP-Rab11 (Fig. 2B). Notably, BIG2 suppression induces a very extensive tubulation of the GFP-Rab11-labelled compartment (Fig. 2B). This is consistent with our time of loading of fluorescent transferrin (30 minutes) which labels primarily recycling (i.e. Rab11-positive) endosomes. The extensive cytosolic background of GFP-Rab11 precludes full quantitative evaluation of tubulation Importantly, tubulation of endosomal compartments was never seen following suppression of BIG1.

### Effect of BIG1 depletion on Golgi organization

**Figure 3:**
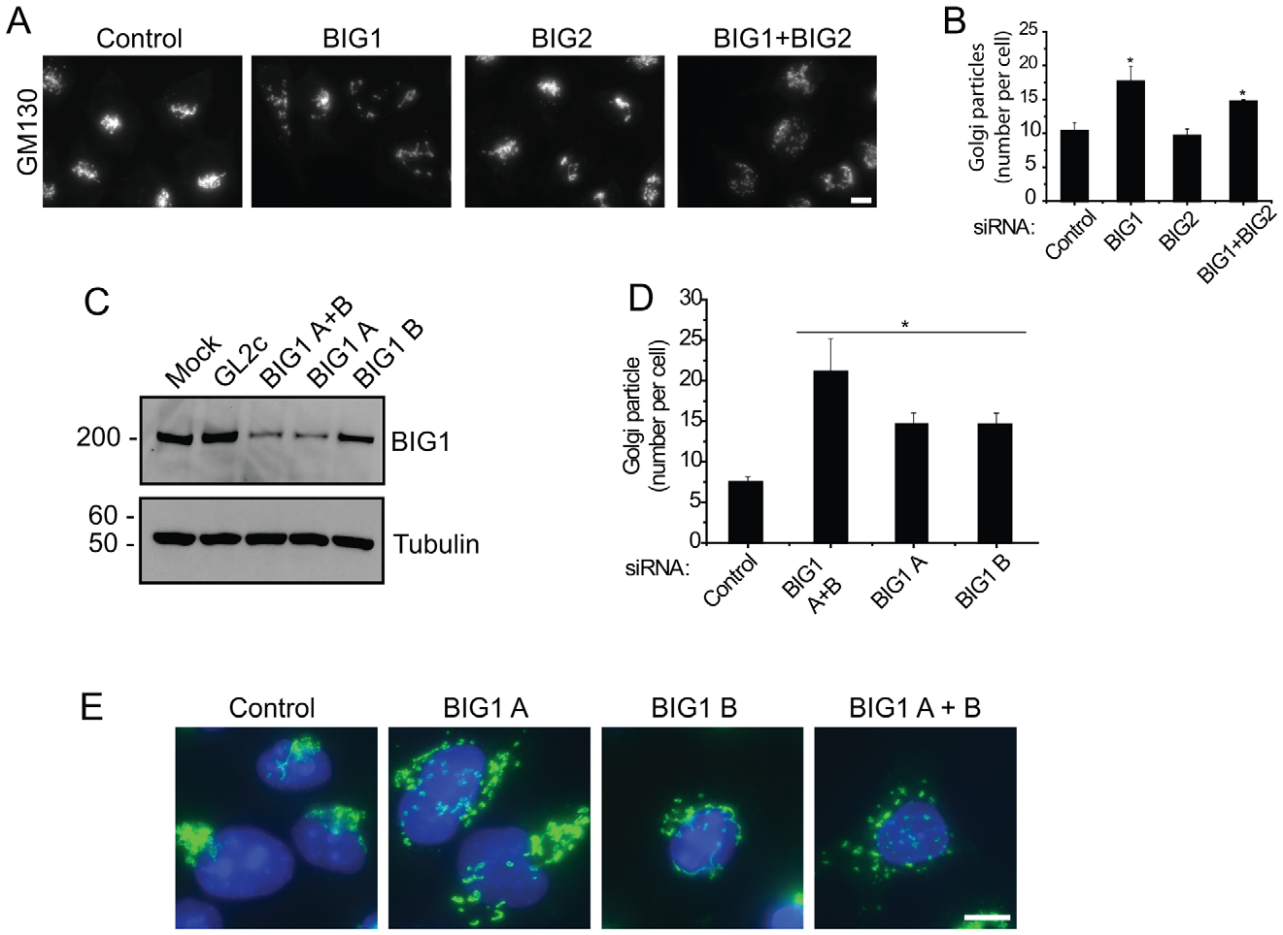
BIG1 suppression induces Golgi fragmentation. **A:** GM130 labelling reveals fragmentation of the Golgi apparatus following suppression of BIG1 or BIG1 and BIG2 together, but control siRNA duplexes or duplexes targeting BIG2 alone do not induce this phenotype. **B:** After applying the same threshold to all images, the number of Golgi fluorescent particles per cell was determined as described in “Materials and Methods”. Results are presented as mean +/− s.e.m., asterisk means p<0.05 as compared to Control-depleted cells, n = 3. **C:** BIG1 suppression using individual siRNA duplexes was monitored by immunoblotting. Tubulin is used as a loading control. **D:** Quantification of Golgi-fragmentation done as in B shows that both individual siRNA give a similar phenotype. **E:** Cells depleted of BIG1 using individual or pooled duplexes were labelled with antibodies against the Golgi matrix protein giantin (in green) and nuclei are counterstained with DAPI (in blue).

As shown in Fig. 3A, and quantified in Fig. 3B, depletion of BIG1 but not of BIG2 induced a fragmentation of the Golgi apparatus. This fragmentation was never observed in BIG2-depleted cells, looking at both cis-Golgi markers GM130 and giantin (Fig. 3A and see also Fig. S1 for a direct comparison). Despite having this profound effect on Golgi organization, BIG1-depletion had no effect on either the Golgi population of COPI or on the punctuate labelling in the cell periphery (Fig. S1, COPI). The lack of effect on Golgi organization following BIG2-depletion could be explained by a compensation of loss of BIG2 function by BIG1. In order to investigate this, we examined cells depleted of both BIG1 and BIG2. Single or double-depleted cells were stained for the Golgi and the number of Golgi fragments per cell quantified. Double-depletions of BIG1 plus BIG2, did not result in a change of the Golgi fragmentation (Fig. 3A, 3B), indicating that they might have different functions in the cell.

To validate these findings further we suppressed BIG1 using our two siRNA duplexes separately (labelled as siRNA A and B). Highly effective suppression of BIG1 expression across a population of cells is seen when using the BIG1 “A” duplex with less effective suppression when using the BIG1 “B” duplex (Fig. 3C). On quantitation of the Golgi fragmentation phenotype, we find that both duplexes cause a significant disruption of Golgi structure (Fig. 3D), evident from giantin labelling (Fig. 3E).

**Figure 4:**
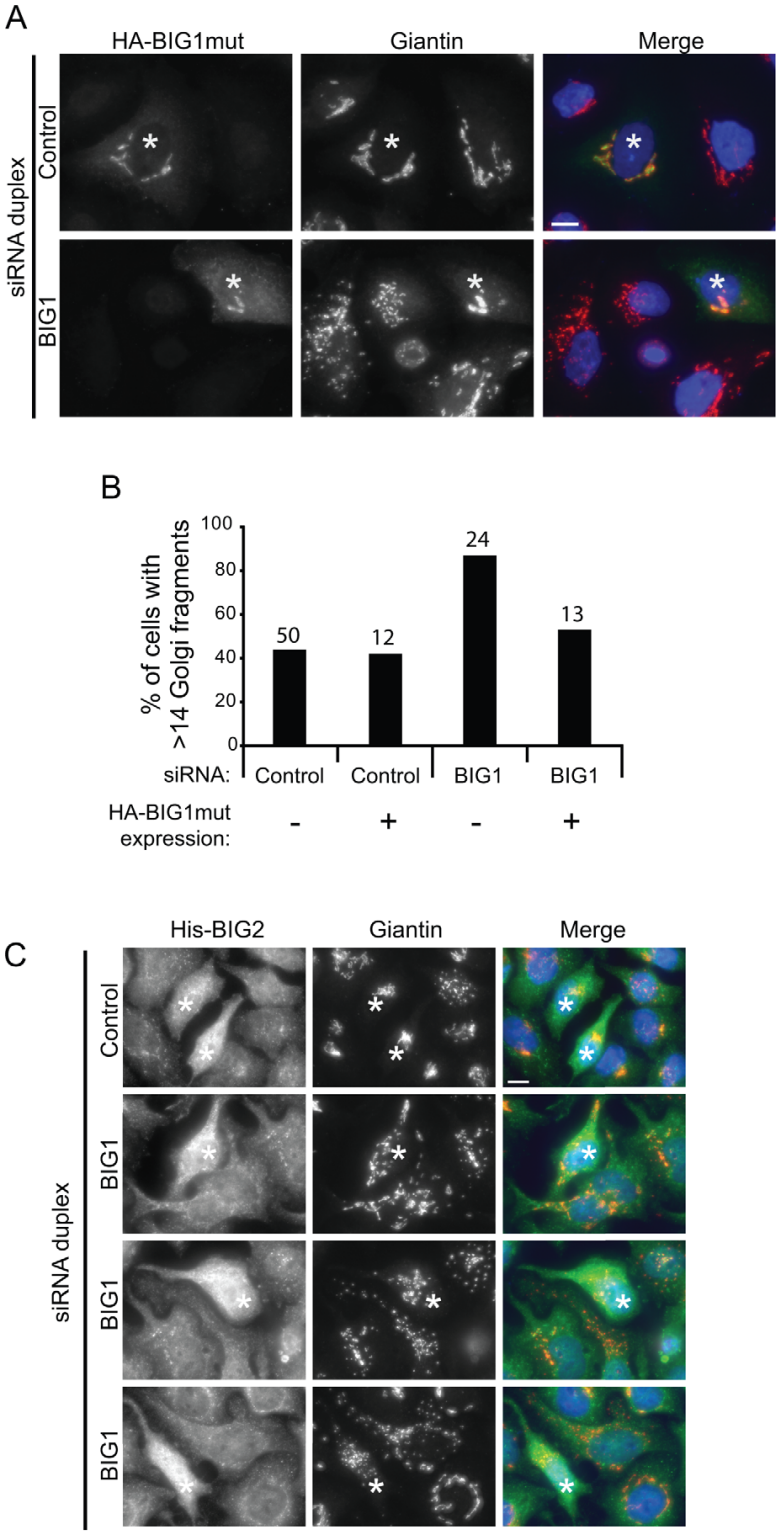
Rescue of Golgi fragmentation in BIG1-suppressed cells. **A:** Control cells, or those depleted of BIG1 expression were transfected to express a HA-tagged mutant BIG1 (HA-BIG1mut) that is resistant to the siRNA. Cells were processed for immunofluorescence using anti-HA (green) and anti-giantin (red) antibodies. Asterisks indicate transfected cells, showing the rescued phenotype in BIG1-depleted cells. **B:** Quantification of the rescue phenotype. Cells were transfected with indicated siRNA and subsequently transfected to express HA-BIG1mut. The number of Golgi particles per cell was counted as in Fig. 3B. Data are pooled from two independent experiments. The numbers on top of the bars indicate the number of cells counted for each condition. **C:** BIG2 expression cannot rescue the fragmentation of the Golgi induced by BIG1-depletion. Control cells or those depleted of BIG1 expression were transfected to express a His-tagged BIG2 (His-BIG2). Cells were labelled using anti-BIG2 (green) and anti-giantin (red) antibodies. Asterisks indicate transfected cells, showing that overexpression of BIG2 does not rescued the phenotype in BIG1-depleted cells. Three different examples are shown for BIG1-depleted cells.

The quantification of the number of Golgi-structures per cell revealed a dramatic fragmentation of the Golgi in BIG1-depleted cells (Fig. 3A, B). The same phenotype was obtained using only single siRNA duplex to deplete BIG1 (Fig. 3C, D, E). In order to ensure the specificity of the BIG1-siRNA phenotype on the Golgi morphology, and to rule out the possible off-target effects of the siRNA duplex, we performed a recovery experiment by expressing a HA-tagged mutant BIG1 cDNA that is resistant to the BIG1 “A” siRNA duplex (HA-BIG1mut). As shown in Fig. 4A, cells expressing HA-BIG1mut after depletion of endogenous BIG1 showed a compact and juxtanuclear Golgi as in control cells consistent with rescue of the siRNA phenotype. Quantification of these data validated the rescue of phenotype (Fig. 4B). It has to be noted that in these experiments the control cells seems to have a looser Golgi (i.e. 40% of cells have more than 14 Golgi particles per cell). An explanation to this could be that those cells have been transfected by siRNA using calcium phosphate transfection and then have been transfected by cDNA using Lipofectamine2000 (see “Materials and Methods”). The cumulative toxicity of both transfections could explain the discrepancy with the quantification in Fig. 3B and 3E). Importantly, overexpression of His-tagged BIG2 could not rescue the Golgi dispersion phenotype in any cells examined (Fig. 4C).

**Figure 5:**
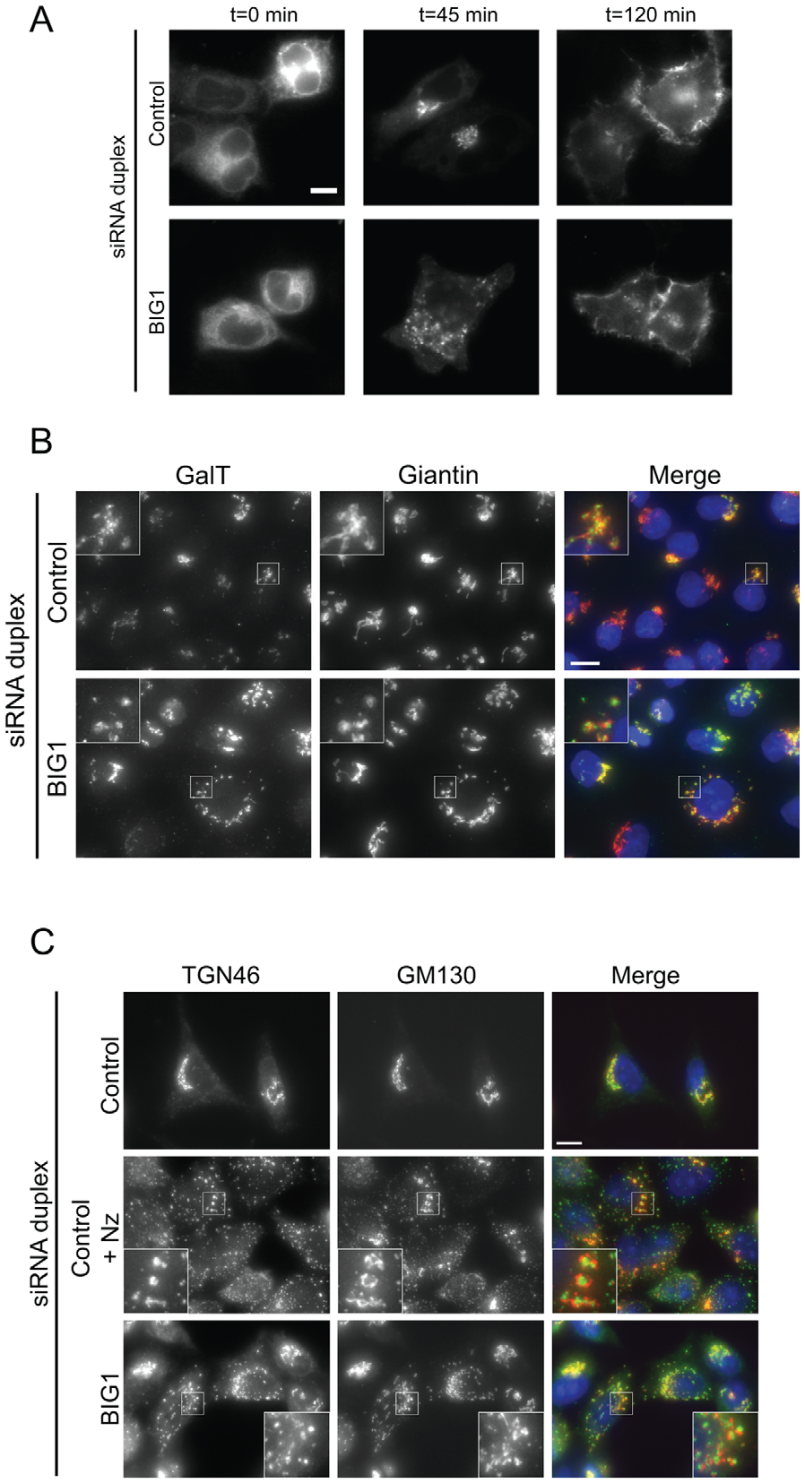
The fragmented Golgi induced by BIG1 suppression remains functional. **A:** Cells were infected with adenovirus to express tsO45-G-YFP at 39.5°C. Cells were then shifted to 32°C to allow the trafficking of the viral protein for 0, 45, or 120 minutes as indicated. Trafficking of the viral protein is indistinguishable between control and BIG1 depleted cells. Note the fragmentation of the Golgi in BIG1 depleted cells confirming the efficacy of BIG1 suppression. **B:** The fragmented Golgi in BIG1-depleted cells is still populated with endogenous galactosyltransferase (GalT). Depleted HeLa cells were processed for immunofluorescence with anti-GalT (green in merge) and anti-giantin (red) antibodies. **C:** Scattered Golgi elements induced by BIG1-depletion retain their cis-trans polarity. HeLa cells transfected with siRNA duplexes as indicated were incubated with nocodazole (Nz) to depolymerize the microtubules, methanol-fixed, and processed for immunofluorescence with anti-TGN46 (green in merge) and anti-GM130 (red) antibodies.

In order to investigate whether Golgi fragmentation caused by BIG1 suppression perturbs the secretory pathway, we first used the model cargo protein tsO45-G-YFP. This glycoprotein can be accumulated in the ER at 39.5°C and then released as a relatively synchronous wave at 32°C [35], [36]. In control cells, tsO45-G-YFP reached the Golgi apparatus after 45 minutes of export, and the plasma membrane within 2 hours (Fig. 5A, Control). In BIG1-depleted cells affected for the Golgi, tsO45-G-YFP was still able to reach the Golgi apparatus and subsequently the plasma membrane (Fig. 5A, BIG1). Gross perturbation of ER-to-Golgi transport results in a redistribution of β1,4-galactosyltransferase (GalT), which is a *trans*-Golgi resident enzyme [37] to the ER [37], [38]. Localization of endogenous GalT was unperturbed in BIG1-depleted cells (Fig. 5B). While clearly functional, we also determined whether these scattered Golgi elements retained the same cis-trans polarity as the Golgi in unperturbed cells. We labelled cells with antibodies directed against GM130, a cis/median-Golgi marker, and TGN46, a well known marker of the TGN. The Golgi mini-stacks obtained in BIG1-depleted cells retained their cis-trans polarity, as is seen after nocodazole treatment in control cells (Fig. 5C, control cells+Nz). It should be noted that the overall expression level of several markers (GM130, Golgin-97, and ERGIC-53) was not altered by any depletion as assessed by western-blot (data not shown). This suggests that the observed fragmentation of the Golgi is not due to a loss of these key matrix proteins, and reinforces the specificity of this phenotype caused by BIG1 depletion.

**Figure 6:**
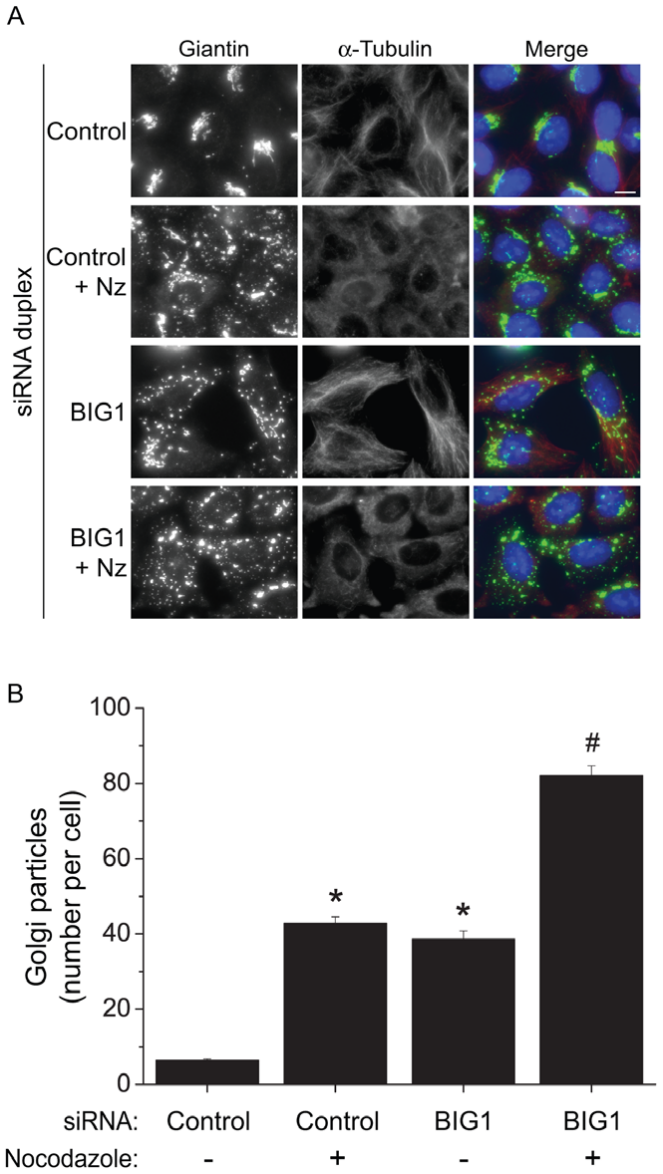
The Golgi mini-stacks in BIG1-depleted cells are still coordinated on microtubules. **A:** HeLa transfected for indicated siRNA were treated or not with nocodazole (Nz) and stained for giantin and α-tubulin. **B:** Quantification of Golgi-particles was done as in previous figures. A higher fragmentation of the Golgi can be achieved by treating BIG1-depleted cells with nocodazole, suggesting that the Golgi mini-stacks are still coordinated on microtubules. Asterisks mean p<0.05 as compared to control untreated cells, # means p<0.05 as compared to nocodazole-treated BIG1-deplepted cells.

In order to investigate if this Golgi fragmentation is due to a general fragmentation of the Golgi or a loss of interaction with the microtubule network, we treated the cells with nocodazole prior to quantification of the Golgi-particles number. As shown in Fig. 6A and quantified in Fig. 6B, when BIG1-depleted cells are treated by nocodazole, a higher level of fragmentation can be achieve compared to untreated depleted cells. This suggests that the fragmentation of the Golgi apparatus in BIG1-depleted cells is not due to a loss of interaction with microtubules but more likely to a break-up of cohesion between Golgi elements with these fragmented ministacks remaining anchored onto the microtubules. It should be noted that the actin cytoskeleton was also unperturbed on BIG1 suppression (Fig. S2B).

This dispersed Golgi phenotype is highly reminiscent of that seen on depletion of Golgi matrix proteins GRASP65 and GM130 [39]. In order to characterize the molecular mechanism causing this Golgi fragmentation, we investigated the recruitment of several Golgi matrix proteins known to be involved in structural maintenance of the Golgi apparatus. Immunofluorescence labelling showed that the peripheral Golgi matrix protein GRASP65 [40], [41] was still associated with the fragmented Golgi (Fig. S3A). Moreover, the antibody used recognizes the C-terminal part of the protein, which is cleaved during apoptotic process leading to the fragmentation of the Golgi [42]. The fact that this antibody detects GRASP65 on the fragmented Golgi together with the normal DAPI staining of the nuclei showed that the fragmentation of the Golgi apparatus in BIG1-depleted cells is therefore not a result of an early apoptotic process. Furthermore, the Golgi matrix proteins p115 (Fig. S3B), GM130 (see Fig. 3A, 5C and S1), giantin (Figs. 3E, 5B, S1 and others), and the TGN-localized protein golgin-97 (data not shown) are still recruited to the fragmented Golgi. BIG1 has been shown to be phosphorylated by PKA and proposed to be an AKAP [25], [26]. The regulatory subunit RIIα of PKA is mainly localized in the juxtanuclear region [43], and recently has been involved in the maintenance of the Golgi apparatus [44]. Fig. S3C shows that the endogenous regulatory subunit RIIα is still present on Golgi membranes following BIG1 depletion. This shows that the maintenance of Golgi structure does not involve the AKAP activity of BIG1, and that BIG1 is not the primary factor maintaining a Golgi pool of PKA.

Clathrin adaptors associated with the TGN are recruited in an Arf-dependent manner [45], [46]. It has been shown that endogenous and overexpressed BIG1 colocalize with the clathrin adaptor AP-1 on the TGN. In our hands, the limited colocalization between AP-1 and TGN46 prevented us from accurately defining the recruitment of AP-1 onto the TGN (Fig. S4A). To address this question, we used HeLa cells stably expressing a CD8-tagged MPR [47] which gives a better colocalization with AP-1. As shown in Fig. S4B, BIG1 depletion did not alter AP-1 localization to the TGN. The peripheral punctate pool of AP-1 was also unchanged, presumably representing peripheral endosomes as described elsewhere [48]. The GGA proteins (GGA1, GGA2, and GGA3) are monomeric clathrin-adaptors that are involved in export of cargo mainly from the TGN to the endosomes [3], [49]. As shown in Fig. S5, the three GGAs were found to be still recruited on the Golgi mini-stacks in BIG1-depleted cells. Similarly, depletion of BIG2 did not affect recruitment of the GGAs to the TGN (data not shown). These data are in accordance with the recent study showing that neither the single depletion of BIG1 or BIG2 nor the double depletions resulted in a loss of GGA proteins from the TGN membranes [34].

**Figure 7:**
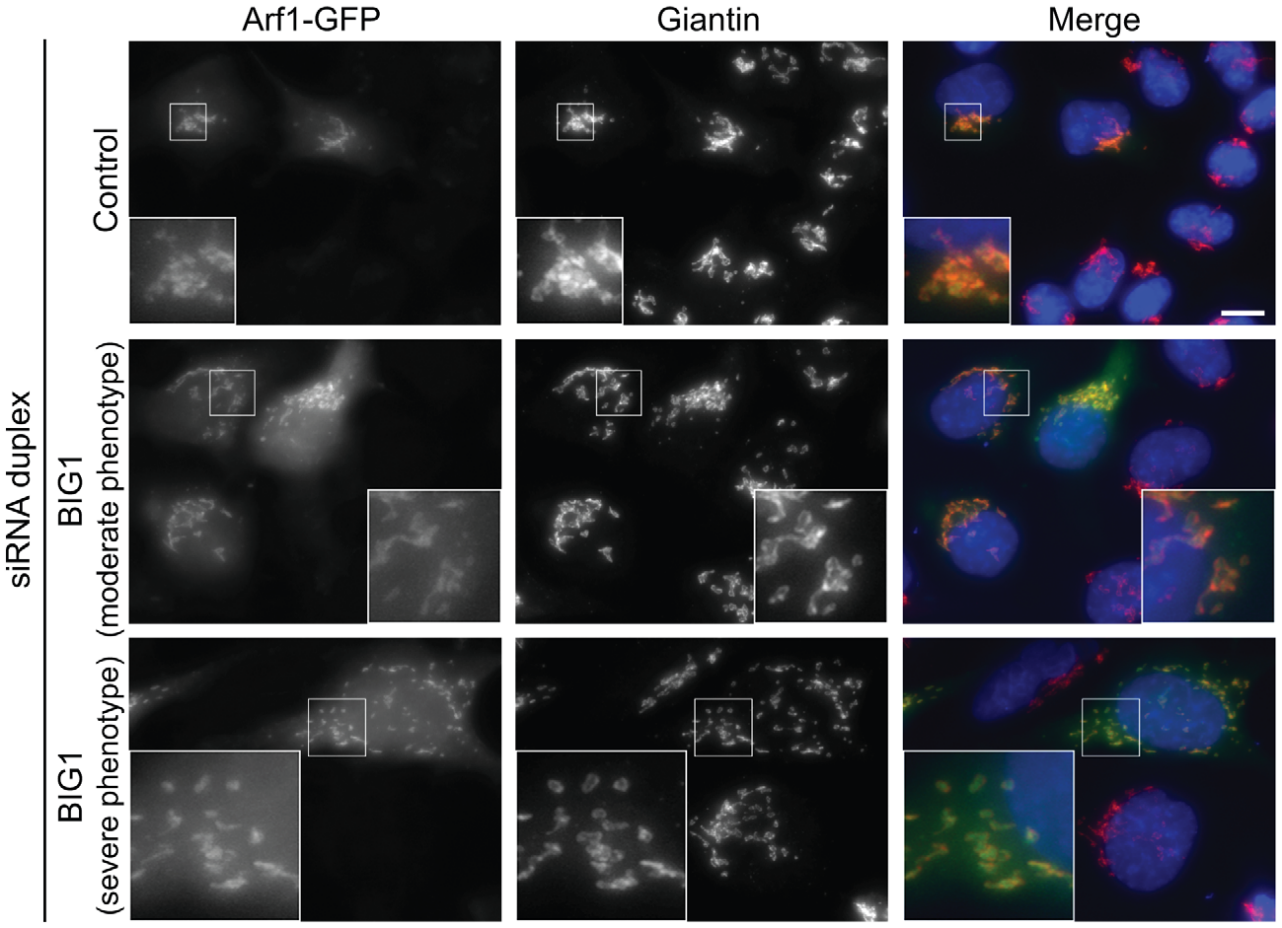
Arf1 is still recruited to Golgi mini-stacks in BIG1-depleted cells. HeLa cells depleted of BIG1 and transiently expressing Arf1-GFP were processed for immunofluorescence with an anti-giantin (red) antibody.

Because these Arf effectors are still recruited to the fragmented Golgi membranes induced by BIG1 depletion, it is tempting to postulate that function of BIG1 in maintaining the structure of the Golgi is independent of its GEF activity. Consistent with this, and as shown in Fig. 7, Arf1-GFP is still recruited to the Golgi fragments in BIG1-depleted cells.

## Discussion

Previous studies of large Arf-GEF function have largely involved the overexpression of mutant forms of these proteins or siRNA-mediated suppression of expression. In most cases examining the roles of BIG1 and BIG2 in particular, the two proteins are treated as redundant and have been suppressed simultaneously in cells. While this clearly eliminates any issues arising from redundancy, such experiments will of course miss any specific roles for these proteins. Furthermore, the expression of dominant negative or constitutively active forms of the large Arf-GEFs could result in formation of complexes that are not physiologically relevant. In our study, we have undertaken a comparative analysis of the two large Arf-GEFs BIG1 and BIG2, targeting each individually. A key goal was to define any non-redundant functions as well as reconcile considerable conflicting data in this field.

### BIG2 is required to maintain recycling endosome structure

Overexpression of a catalytically inactive mutant of BIG2 induces the formation of tubules which are positive for several markers of the recycling endosomes (Rab4, Rab11 and transferrin-receptor) and negative for the TGN marker TGN46 and early as well late endosomes/lysosomes (Rab5, Rab7, EEA1 and LAMP1) [21]. In our hands siRNA depletion of BIG2 recapitulates these effects, inducing the tubulation of recycling endosomes positive for Rab11-GFP. Together these data indicate that endosomal tubulation is a specific phenotype resulting from BIG2 perturbation and that endosome morphology cannot be compensated for by BIG1. In contrast, while previous work has shown that suppression of both BIG1 and BIG2 results in loss of AP1 from TGN membranes, we find that suppression of BIG1 alone does not affect AP1 recruitment consistent with a non-redundant function of BIG1 and BIG2 at the TGN [34].In agreement with other findings, suppression of BIG1 does not result in a loss of GGA from membranes.

### A specific role for BIG1 in the maintenance of Golgi structure

The key finding from our work is that suppression of expression of BIG1 results in a fragmentation of the Golgi apparatus. This phenotype is similar to one previously described [20], but the effect was only detectable by electron microscopy. Previous double depletion of BIG1 and BIG2 resulted in the dispersion of the TGN with no apparent changes in the Golgi morphology [50]. A potential explanation for this would be an incomplete suppression of BIG1 expression in these experiments. These data [50] and others [34] do however suggest redundant functions of BIG1 and BIG2 at the TGN. Our data show that maintenance of Golgi structure is one function of BIG1 that cannot be compensated for by BIG2. Despite retention of Arf1-GFP at the Golgi, it is possible that other Arf proteins fail to be recruited to the Golgi mini-stacks in BIG1-depleted cells. Another interpretation of this is that the role of BIG1 in maintaining the integrity of the Golgi apparatus is independent of its function as an Arf-GEF.

The Golgi fragmentation phenotype that we observe is distinct from that seen with nocodazole treatment in that the fragments are larger and do not become scattered throughout the cytoplasm, in addition we observe no changes in the integrity of the microtubule cytoskeleton following BIG1 suppression. Moreover, nocodazole-treatment of BIG1-depleted cells highly suggests that the Golgi mini-stacks are still organized by microtubules. The lack of correlation between the level of BIG1 knockdown and the extent of Golgi fragmentation could be indicative of a threshold having been reached beyond which one sees no further phenotype. This would be consistent with the results following addition of nocodazole. This phenotype is highly reminiscent of that seen following suppression of expression of Golgi matrix proteins [39]. Despite this, we did not detect any perturbation in the localization to the Golgi of, or stability of GM130, GRASP65, giantin, or p115. BIG1 interacts with myosin IXb [51]. Little is known of the function of myosin IXb but it has been localized throughout the cytoplasm and on the Golgi apparatus. It contains a RhoGAP domain and it is tempting to postulate that myosin IXb is needed for the maintenance of the Golgi apparatus through integration of Golgi membranes with the actin cytoskeleton.

A further point from our experiments is that we find that BIG1 is not absolutely required for the recruitment of GGAs to membranes. This is likely again due to redundancy with other Arf-GEFs, possibly BIG2 [50] but also somewhat surprisingly, GBF1 [52]. This reinforces the difficulty in assigning specific function to these Arf-GEFs and underlies the specificity of the Golgi fragmentation phenotype on suppression of BIG1.

## Materials and Methods

### Materials

Nocodazole was from Sigma, Alexa568-conjugated phalloidin and transferrin from Invitrogen. Antibodies used were as followed: mouse monoclonal anti-GBF1 (BD Transduction Laboratories); rabbit polyclonal anti-BIG1 and anti-BIG2 (Bethyl Laboratories); monoclonal mouse anti-α-tubulin DM1A (Labvision); polyclonal rabbit anti-COPII (Sec24C) was as described [53]; monoclonal mouse anti-EEA1 (BD Transduction Laboratories); monoclonal anti-AP-1 (Sigma); monoclonal anti-GGA3 (BD Transduction Laboratories); polyclonal anti-COPI (BSTR) [38]; rabbit polyclonal anti-GGA1 and anti-GGA2 were kindly provided by Jennifer Hirst (CIMR, Cambridge, UK); rabbit polyclonal anti-giantin (Covance); monoclonal mouse anti-GM130 (BD Transduction Laboratories); polyclonal sheep anti-TGN46 (AbD Serotec); monoclonal mouse anti-RIIα PKA (BD Transduction Laboratories); monoclonal mouse anti-β1,4-galactosyltransferase (CellMab AB); polyclonal sheep anti-GRASP65 and monoclonal mouse anti-p115 were kindly provided by Jon Lane (University of Bristol, Bristol, UK); polyclonal rat and monoclonal mouse anti-CD8 were kindly provided by Pete Cullen (University of Bristol, Bristol, UK) as well as plasmid encoding for GFP-Rab11. Cy-dye and HRP-conjugated secondary antibodies were from Jackson Immunoresearch. Monoclonal mouse anti-CD63 (Biogenesis) was kindly provided by Harry Mellor (University of Bristol, Bristol, UK). Plasmid encoding Arf1-GFP was kindly provided by Jennifer Lippincott-Schwartz [54]. HeLa cells stably expressing CD8-MPR were a kind gift from Matthew Seaman (CIMR, Cambridge, UK) [47].

### Molecular cloning and site-directed mutagenesis

To obtain a siRNA-resistant form of BIG1, a HA-tagged version kindly provided by M. Vaughan [55] was used as a template for site-directed mutagenesis using the following sense primer: 5′- GGAACAAAAGATCAGGC(T/A)CC(T/A)GA(T/C)GAATT(T/C)GTGGGTTTAGGGC -3′ in combination with its antisense primer. The nucleotides in brackets indicate the bases that were changed to produce base substitutions T3261A, T3264A, T3267C and T3273C, according to the human ORF for BIG1. The base substitutions also produce an additional EcoRI restriction site (underline sequence) to allow simple screening of colonies for mutant plasmids. The resulting construct was fully sequenced to ensure the fidelity of the PCR. Only a single mutation was found (C1670T), resulting in a A557F change in the amino acid sequence.

### Tissue culture, transfection and immunoblotting

HeLa cells (ATCC CCL-2) were grown as described before [56]. For each large GEF, two siRNA duplexes were designed and ordered (MWG Biotech) as follows (sense oligonucleotide sequences): BIG1 (“A” AGAUCAGGCUCCUGAUGAATT; “B” ACAGAGUCCUCCUCAUGGATT); BIG2 (“A” UACUGAACAUAGCUGUAUATT; “B” AGGCCUUCAACUCCAAUUATT). As a targeted control, two different unrelated siRNA were used in this study: lamin A/C [56] or GL2c directed against firefly luciferase [57] (CGUACGCGGAAUACUUCGATT). Both control oligonucleotides gave undistinguishable absence of effect on all the organelles looked at and are referred in this study as the “Control”. Cells were transfected as described [56] using calcium phosphate for siRNA or FuGENE6 (Roche Applied Science) or Lipofectamine™ 2000 (Invitrogen) for DNA. To confirm the depletion by immunoblotting, cells were washed twice in ice-cold PBS, scrapped in ice-cold buffer A [50 mM Tris-HCl, 150 mM NaCl, 1% Triton X-100, Protease Inhibitor Cocktail Set V (Calbiochem), pH 7.4], left on ice for 30 minutes, insoluble material was pelleted by centrifugation (10,000 g for 10 minutes at 4°C). The protein concentration was assayed using BCA™ Protein Assay Kit (Pierce/Thermo Fisher) according to the manufacturer's instructions. Same amount of proteins was diluted in SDS-PAGE sample buffer, denatured at 70°C for 10 minutes, and proteins were separated by electrophoresis on Tris-Acetate or Bis-Tris gels (Invitrogen), transferred to nitrocellulose and immunoblotted. Blots were developed using ECL (GE Healthcare).

### Immunofluorescence and live cell imaging

Living and fixed cells were imaged by using widefield microscopy as previously described [56]. If not stated differently, all images shown are extended focus through sample. Where indicated, cells were treated with nocodazole (5 µM) or with an equal volume of DMSO (vehicle only) for 1 h in culture medium. For immunofluorescence, cells were fixed with methanol for 4 minutes at −20°C, blocked with using PBS containing 3% bovine serum albumin and incubated with primary antibodies for 1 hour. For staining of EEA1, cells were fixed with 3.5% paraformaldehyde for 15 minutes and permeabilized with 0.1% Triton X-100 in PBS for 5 minutes. For Alexa568-transferrin uptake, HeLa cells were grown on glass-bottom dishes (MatTek Corp.) and Alexa568-transferrin was diluted in imaging medium (DMEM without phenol red supplemented by 20 mM HEPES pH 7.4) at a final concentration of 100 µg.ml^−1^. Images and time-lapse sequences were acquired at 37°C, 30 minutes after Alexa568-transferrin incubation.

### tsO45-G-YFP transport assay

Quantitative transport assay of tsO45-G-YFP was essentially done as described before [56]. Briefly, depleted cells were infected with Adenovirus engineered to express tsO45-G-YFP for 1 hour at 37°C, and then washed and transferred to 39.5°C for 16 hours. Transport assays were carried out by shifting cells to 32°C for the times indicated. The cells were then paraformaldehyde-fixed and proceeded for immunofluorescence as described above.

### Image and statistical analysis

All images were processed using Photoshop CS (Adobe), and ImageJ. Movies were compiled in QuickTime Pro using “Photo JPEG” compression. For quantitative analysis of the Golgi/TGN fragmentation, the same threshold was applied to all images, and the number of Golgi-derived particles per cell was determined using the ‘Analyze Particles’ function of the ImageJ software, as described elsewhere [44]. Between 50 and 200 cells were counted for each condition, and all results from 3–7 independent experiments were pooled and are presented as means +/− s.e.m. Statistical analysis was performed by Student's two-tailed t-test for unpaired data and a probability level of 0.05 was considered significant.

## Supporting Information

Figure S1 Subcellular compartments morphology in BIG1 or BIG2-depleted cells. HeLa cells were depleted with indicated siRNA duplexes, fixed and proceeded for immunofluorescence with antibodies against COPII, EEA1, COPI, Giantin or GM130. Bar = 10 µm in all figures.
(2.12 MB TIF)

Figure S2 The cytoskeleton is not altered in BIG-depleted cells. HeLa cells depleted by indicated siRNA duplexes were stained using antibodies against Giantin and against α-tubulin (panel A) or labelled with Alexa568-phalloidin (panel B).
(2.02 MB TIF)

Figure S3 Recruitment of Golgi proteins to the Golgi-mini-stacks is not impaired in BIG1-depleted cells. Control or BIG1-depleted cells were subjected to immunofluorescence for indicated antibodies. GRASP65, PKA-RIIa and p115 are still localized to the mini-stacks co-labelled with giantin.
(3.61 MB TIF)

Figure S4 BIG1 function on the Golgi maintenance in independent of the recruitment of AP-1. A: Depleted cells treated or not with nocodazole (Nz) were immunolabelled with an anti-TGN46 antibody (green in merge) and with an anti-AP-1 antibody (red in merge). B: HeLa cells stably expressing a CD8-tagged MPR were stained with an anti-CD8 antibody (green in merge) and an anti-AP-1 antibody (red in merge).
(5.77 MB TIF)

Figure S5 Recruitment of GGAs is not affected by BIG1-depletion. HeLa cells expressing CD8-MPR were treated as in Fig. S3 and labelled with an anti-CD8 antibody (green) and with anti-GGA1, GGA2 or GGA3 antibodies as indicated (red in merge).
(4.44 MB TIF)

Movie S1 Depletion of BIG2 induces the formation of stable endosomal tubules. HeLa cells depleted for BIG2 were loaded with Alexa568-conjugated transferrin for 30 minutes and images using wide-field time microscopy. Note some vesicles moving alongside the tubules (most evident on the top-left corner).
(4.14 MB MOV)
