# Supplementary figures and images for: Specific Functions of BIG1 and BIG2 in Endomembrane Organization
Source: PLoS One. 2010 Mar 25;5(3):e9898. doi: 10.1371/journal.pone.0009898 (PMC2845624; doi:10.1371/journal.pone.0009898)

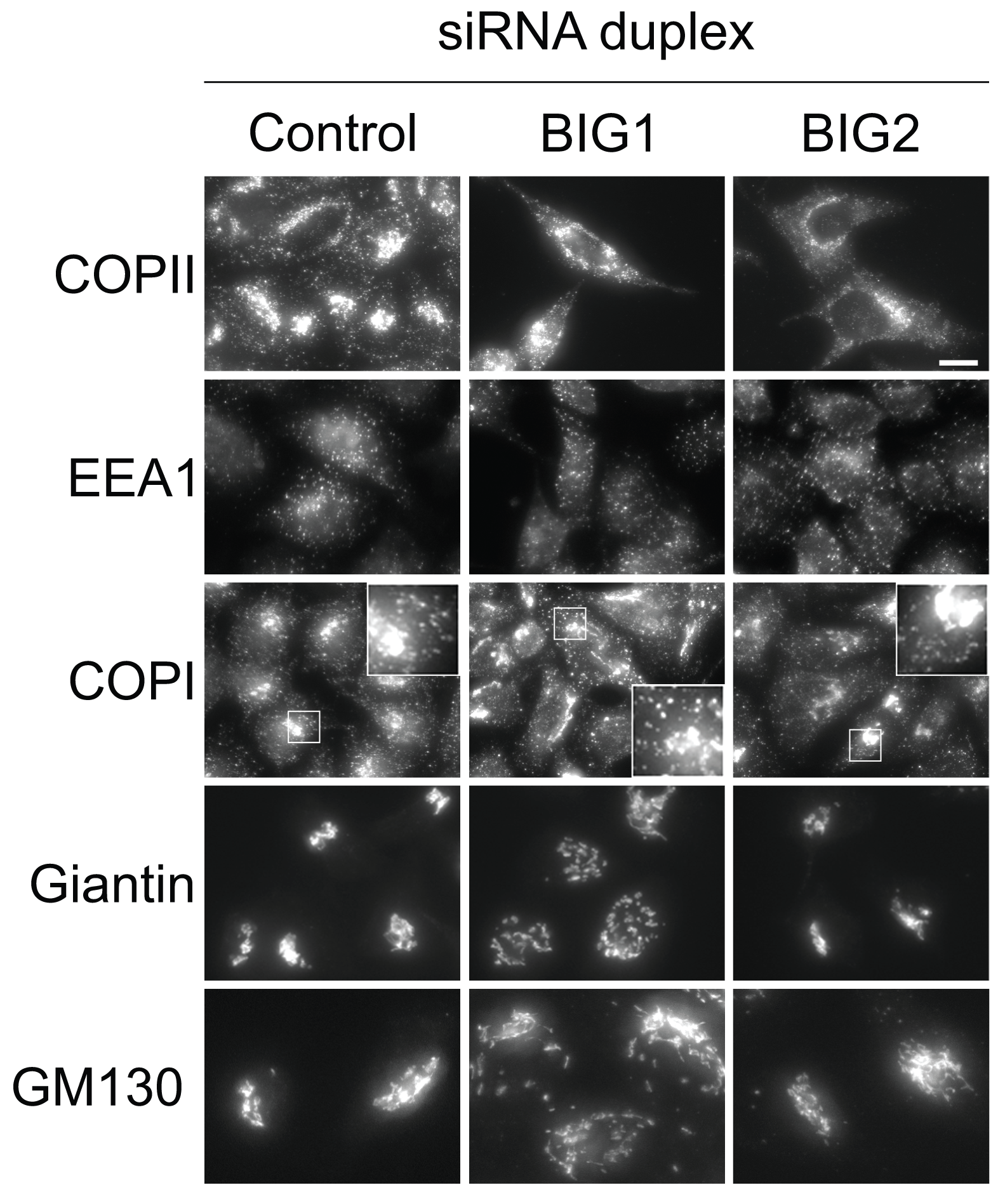

Supplement: Figure S1 — Subcellular compartments morphology in BIG1 or BIG2-depleted cells. HeLa cells were depleted with indicated siRNA duplexes, fixed and proceeded for immunofluorescence with antibodies against COPII, EEA1, COPI, Giantin or GM130. Bar = 10 µm in all figures. (2.12 MB TIF) [file pone.0009898.s001.tif]

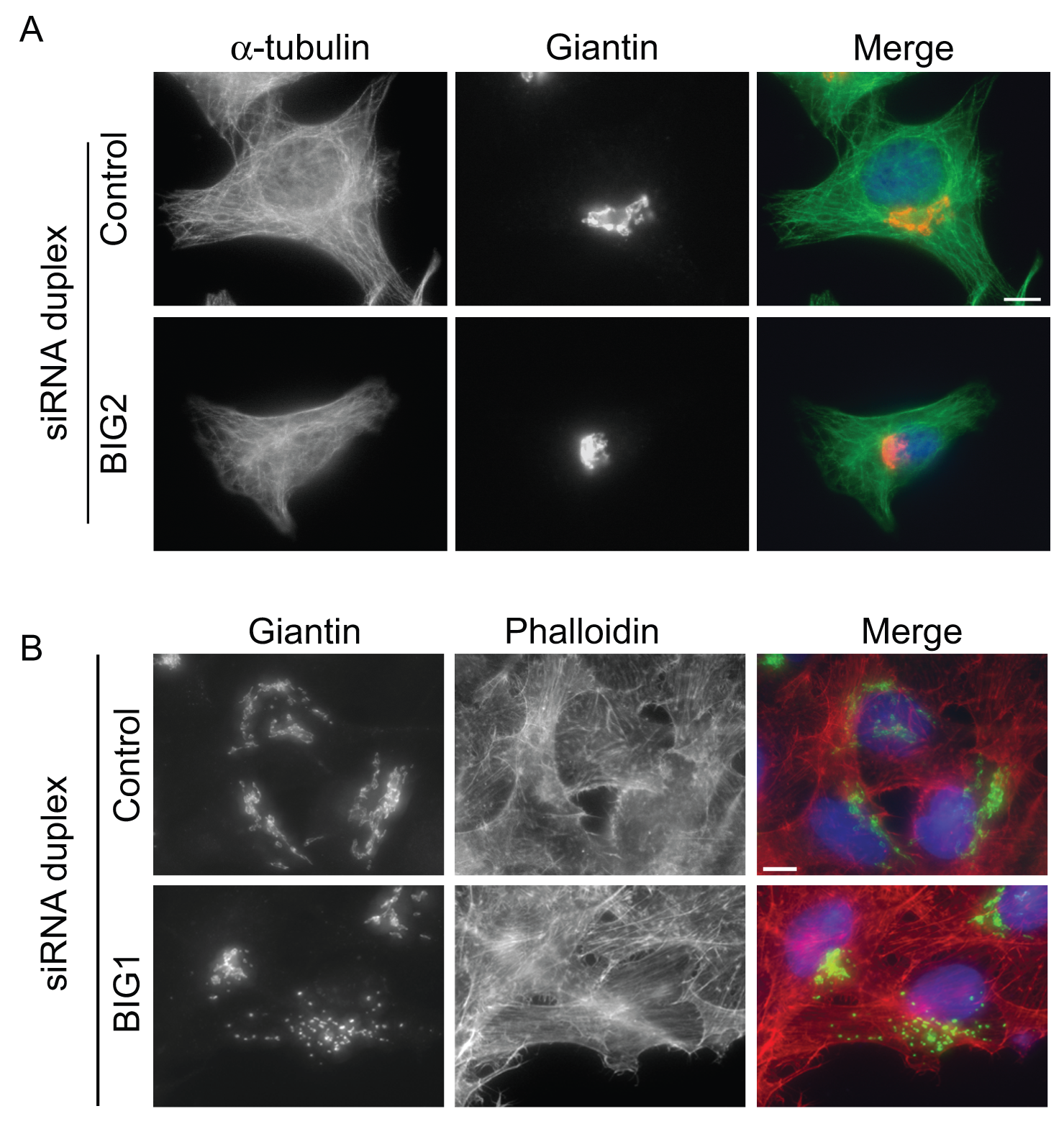

Supplement: Figure S2 — The cytoskeleton is not altered in BIG-depleted cells. HeLa cells depleted by indicated siRNA duplexes were stained using antibodies against Giantin and against α-tubulin (panel A) or labelled with Alexa568-phalloidin (panel B). (2.02 MB TIF) [file pone.0009898.s002.tif]

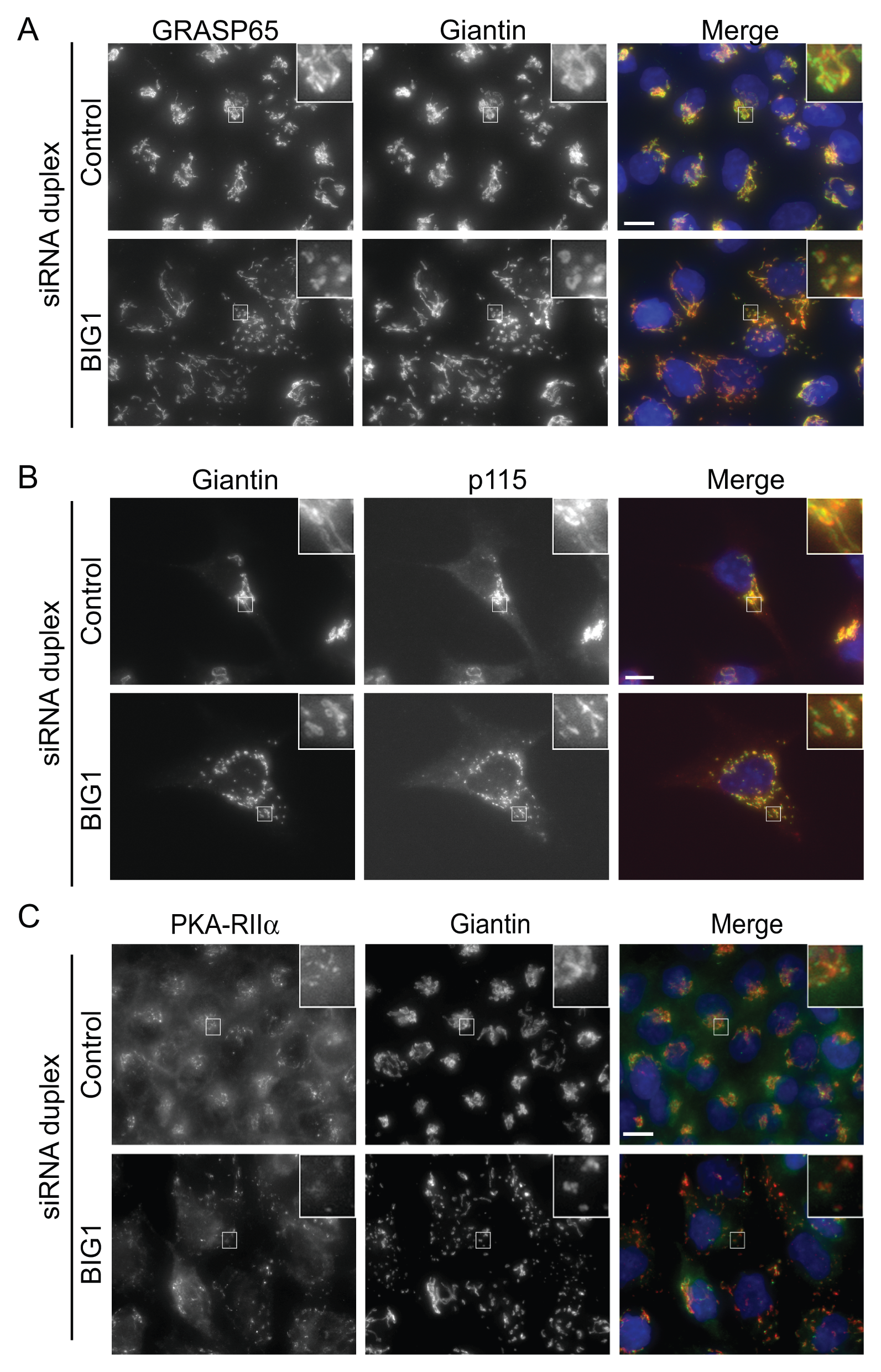

Supplement: Figure S3 — Recruitment of Golgi proteins to the Golgi-mini-stacks is not impaired in BIG1-depleted cells. Control or BIG1-depleted cells were subjected to immunofluorescence for indicated antibodies. GRASP65, PKA-RIIa and p115 are still localized to the mini-stacks co-labelled with giantin. (3.61 MB TIF) [file pone.0009898.s003.tif]

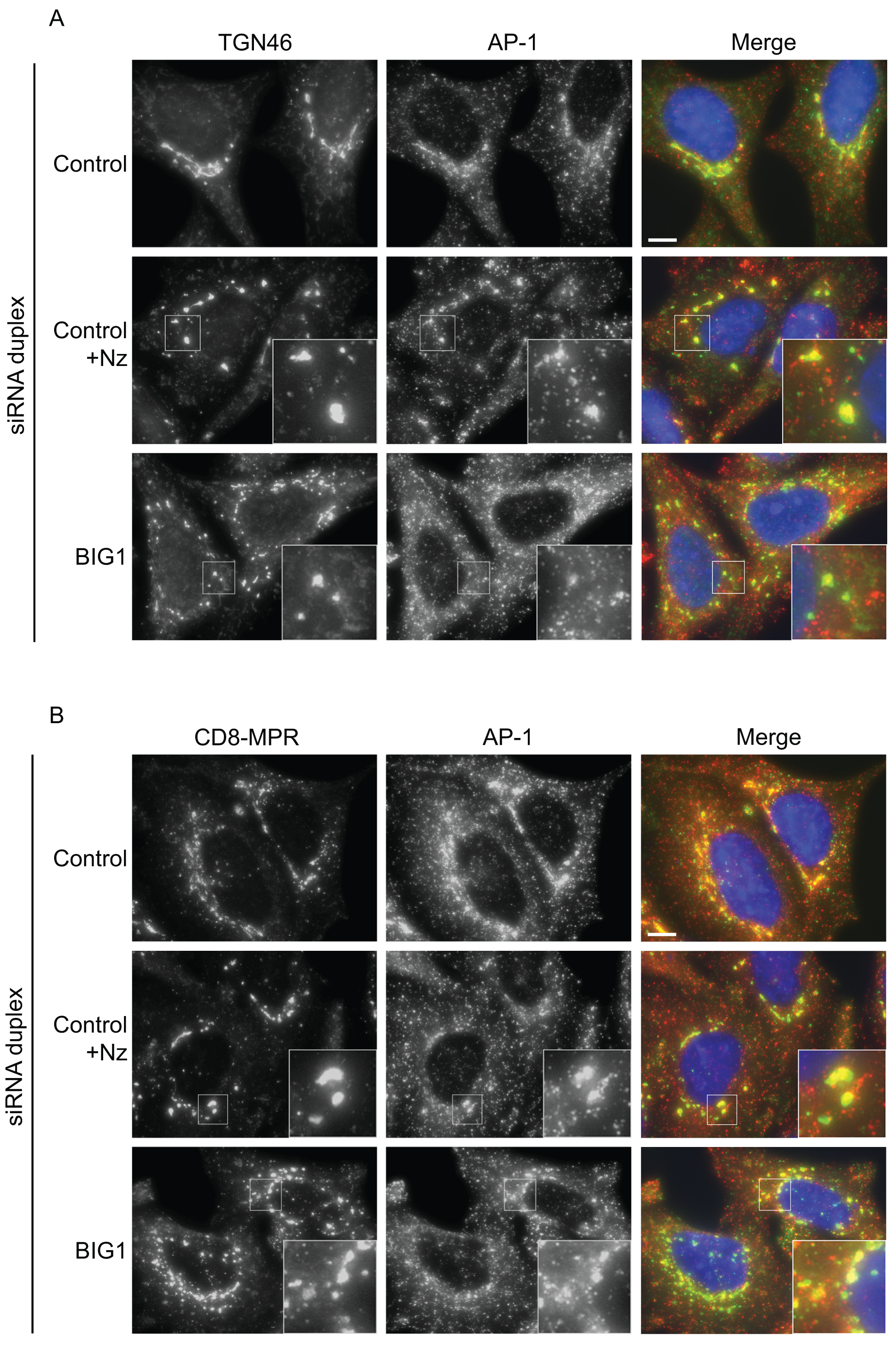

Supplement: Figure S4 — BIG1 function on the Golgi maintenance in independent of the recruitment of AP-1. A: Depleted cells treated or not with nocodazole (Nz) were immunolabelled with an anti-TGN46 antibody (green in merge) and with an anti-AP-1 antibody (red in merge). B: HeLa cells stably expressing a CD8-tagged MPR were stained with an anti-CD8 antibody (green in merge) and an anti-AP-1 antibody (red in merge). (5.77 MB TIF) [file pone.0009898.s004.tif]

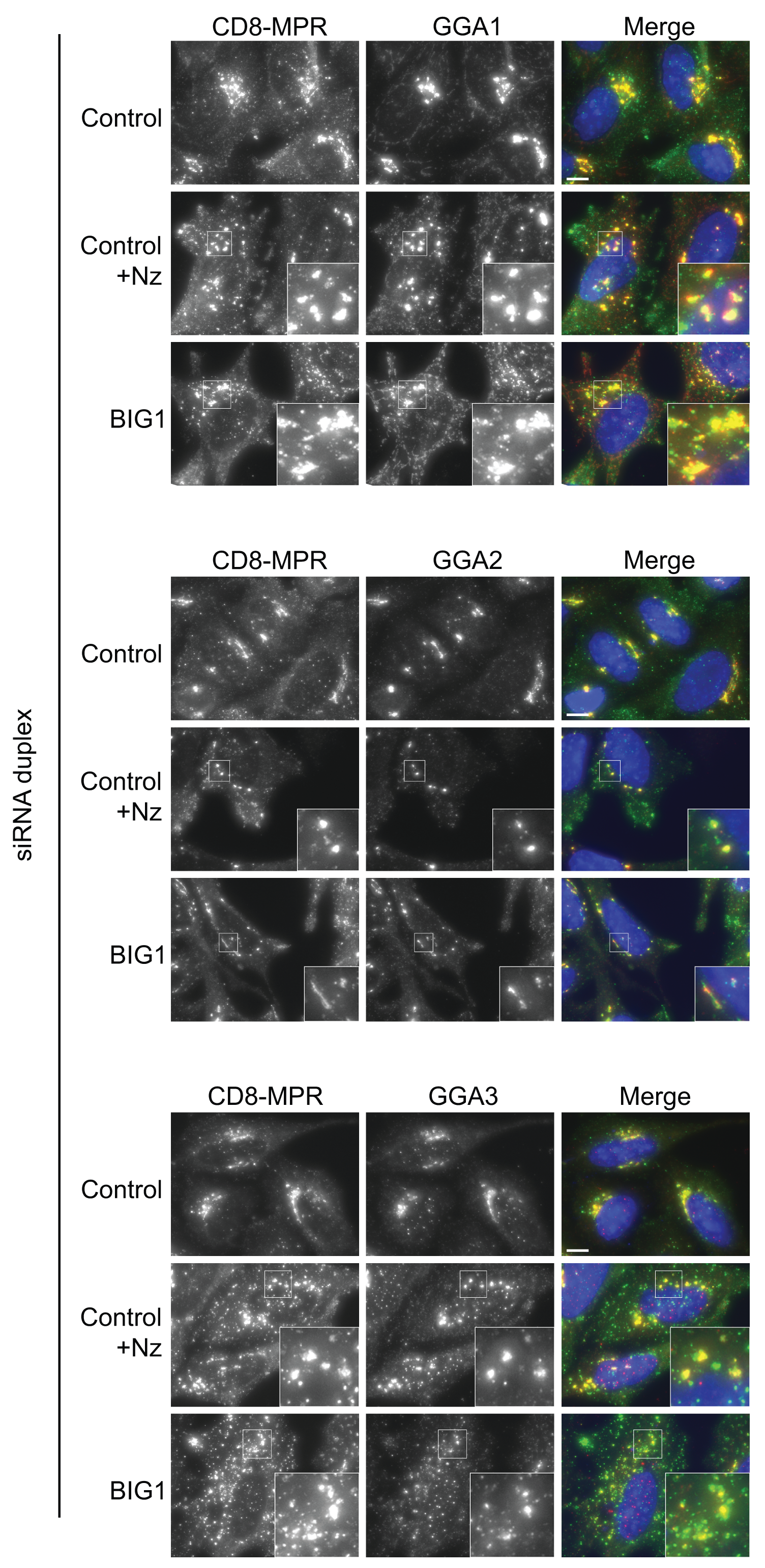

Supplement: Figure S5 — Recruitment of GGAs is not affected by BIG1-depletion. HeLa cells expressing CD8-MPR were treated as in Fig. S3 and labelled with an anti-CD8 antibody (green) and with anti-GGA1, GGA2 or GGA3 antibodies as indicated (red in merge). (4.44 MB TIF) [file pone.0009898.s005.tif]
